# A Framework for Characterizing eHealth Literacy Demands and Barriers

**DOI:** 10.2196/jmir.1750

**Published:** 2011-11-17

**Authors:** Connie V Chan, David R Kaufman

**Affiliations:** ^1^Biomedical InformaticsColumbia UniversityNew York, NYUnited States

**Keywords:** eHealth, health literacy, cognition, Bloom’s taxonomy, cognitive task analysis, consumer health

## Abstract

**Background:**

Consumer eHealth interventions are of a growing importance in the individual management of health and health behaviors. However, a range of access, resources, and skills barriers prevent health care consumers from fully engaging in and benefiting from the spectrum of eHealth interventions. Consumers may engage in a range of eHealth tasks, such as participating in health discussion forums and entering information into a personal health record. eHealth literacy names a set of skills and knowledge that are essential for productive interactions with technology-based health tools, such as proficiency in information retrieval strategies, and communicating health concepts effectively.

**Objective:**

We propose a theoretical and methodological framework for characterizing complexity of eHealth tasks, which can be used to diagnose and describe literacy barriers and inform the development of solution strategies.

**Methods:**

We adapted and integrated two existing theoretical models relevant to the analysis of eHealth literacy into a single framework to systematically categorize and describe task demands and user performance on tasks needed by health care consumers in the information age. The method derived from the framework is applied to (1) code task demands using a cognitive task analysis, and (2) code user performance on tasks. The framework and method are applied to the analysis of a Web-based consumer eHealth task with information-seeking and decision-making demands. We present the results from the in-depth analysis of the task performance of a single user as well as of 20 users on the same task to illustrate both the detailed analysis and the aggregate measures obtained and potential analyses that can be performed using this method.

**Results:**

The analysis shows that the framework can be used to classify task demands as well as the barriers encountered in user performance of the tasks. Our approach can be used to (1) characterize the challenges confronted by participants in performing the tasks, (2) determine the extent to which application of the framework to the cognitive task analysis can predict and explain the problems encountered by participants, and (3) inform revisions to the framework to increase accuracy of predictions.

**Conclusions:**

The results of this illustrative application suggest that the framework is useful for characterizing task complexity and for diagnosing and explaining barriers encountered in task completion. The framework and analytic approach can be a potentially powerful generative research platform to inform development of rigorous eHealth examination and design instruments, such as to assess eHealth competence, to design and evaluate consumer eHealth tools, and to develop an eHealth curriculum.

## Introduction

eHealth literacy names a set of skills and knowledge that are essential for productive interactions with technology-based health tools. The objective of this study was to explore how eHealth literacy can be systematically analyzed, measured, and quantified. We proposed a methodological and theoretical framework that systematically maps skill sets to successful performance of eHealth tasks. We employed a microanalytic strategy in which complex competencies can be broken down into constituent skills or local task demands. In our view, systematic understanding of the necessary competencies can inform development of targeted solution strategies to overcome skill- and knowledge-related barriers.

### Background

The term eHealth refers to “health services and information delivered or enhanced through the Internet and related technologies” [1]. Consumer-oriented eHealth tools engage consumers in managing their own health care, communicating with providers and support networks, meeting their information needs, making health decisions, using patient education resources, and promoting healthy lifestyles [2-4]. Unfortunately, most of these eHealth tools have not been designed with the consideration of the needs and characteristics of diverse user groups. These tools may even increase the complexity of health care engagement for those lacking the prerequisite abilities [5].

Many different factors can inhibit consumers’ meaningful use of eHealth tools, including environmental barriers [6], physical access barriers [7], resource-related barriers [8-11], and individual-level barriers [2,7,12,13]. Underserved and vulnerable populations face additional challenges that exacerbate these obstacles [14]. Different types of tools offer varied resources and functionalities, enabling performance on a wide range of eHealth tasks. Hence, different types of challenges arise depending on the tool. Specifically, interaction with different eHealth tools and tasks makes different kinds of demands on skills and knowledge. Table 1 [11,15-19] lists some examples of documented skill-related challenges that may lead to barriers to the use of different eHealth tools.

**Table 1:** Documented skill-related challenges to use of common eHealth tools

eHealth tool	Example of tasks	Examples of skill-related challenges in completing eHealth tasks
Health information portals	Looking up information about treatment options for a health condition	Identifying appropriate and reliable sources; assessing quality of information
		Using effective information retrieval strategies [15]
		Understanding complex technical language
		Comprehending materials written above recommended reading levels [11]
Personal health records	Entering personal information into medical record	Having computer skills to effectively use all the different features and tools
		Being familiar with health concepts to enter and extract appropriate information in record [16]
Telemedicine or teleconsultation applications	Communicating with health care providers	Effectively using communication tools
		Interpreting and using health information appropriately for self-care activities [17]
Decision-support tools	Evaluating and weighing evidence to inform a decision	Understanding risk and uncertainty [18]
		Obtaining and evaluating evidence-based information
Online support or chat groups	Participating in discussion forum	Communicating ideas clearly; adhering to online social etiquette and group norms
		Effectively sharing information without compromising one’s privacy [19]

There is a divide between what consumers can reasonably be expected to do and the demands and available resources of different tools. Various research efforts, in areas such as educational media, health literacy, and numeracy research, have tried to bridge this gulf by addressing user knowledge and competence, and improving resources. Addressing access and skills barriers has helped underserved and vulnerable populations to use technology in terms of managing their health concerns [20,21]. Therefore, it is important to identify barriers and devise solution strategies to eliminate obstacles that reinforce eHealth disparities.

Our approach is an effort to make this a more tractable problem by identifying candidate explanatory constructs and employing cognitive task analysis (CTA) methods to new applications. To the best of our knowledge, this is a unique effort to introduce systematicity to this complex and ill-defined research space. In our view, the success of consumer health informatics initiatives is partially predicated on an understanding of eHealth literacy demands and competencies.

### Theoretical Framework

In this research, we endeavored to develop a systematic approach to analyzing competencies across eHealth interventions. The objective of this research was to understand the core skills and knowledge needed to productively use eHealth tools and to develop a set of methods for analyzing eHealth literacy. Previously, we presented a preliminary sketch of our framework for characterizing eHealth literacy task demands [22]. In this study, we explored further application of the framework to characterize human task performance.

The approach draws on two established models: the eHealth literacy model and Bloom’s taxonomy of educational objectives. eHealth literacy is defined as “the ability to seek, find, understand, and appraise health information from electronic sources and apply the knowledge gained to addressing or solving a health problem” [23]. The eHealth literacy model describes the set of “fundamental skills consumers require to derive direct benefits from eHealth” [23]. This set of skills establishes an important starting point but does not provide us with a means to discern how different cognitive functions or processes are engaged by different tasks. In addition, eHealth skills may be acquired in different stages, and thus individuals may display different degrees of competence in these skills. We incorporated a second model that is designed to discriminate between various kinds of cognitive processes and describe dimensions of cognitive complexity. Bloom’s taxonomy describes the increasing progression in complexity of cognitive aspects of learning, skill acquisition, and performance, and it has been applied to a range of different topic domains [24-26]. Incorporating this model allows us to characterize the central cognitive processes that constitute each literacy type. The eHealth literacy model defines a literacy type or content domain, while the cognitive taxonomy provides a means of realizing how this can be expressed in the context of task performance. 

In our framework, we adapted the eHealth literacy model proposed by Norman and Skinner in 2006 as a point of departure. Their model describes six components of eHealth literacy [23]:

- *Computer literacy* describes a wide range of skills from basic knowledge of using a computer, such as opening a browser window, to participating in social networking activities.
- *Information literacy* encompasses the skills to articulate information needs, to locate, evaluate, and use information, and to apply information to create and communicate knowledge [27].
- *Media literacy* is the ability to select, interpret, evaluate, contextualize, and create meaning from resources presented in a variety of visual or audio forms [28].
- *Traditional literacy and numeracy* encompasses reading and understanding written passages, communicating and writing a language coherently, quantitative skills, and the ability to interpret information artifacts such as graphs, scales, and forms [29,30].
- *Science literacy* includes familiarity with basic biological concepts and the scientific method, as well as the ability to understand, evaluate, and interpret health research findings using appropriate scientific reasoning [31].
- *Health literacy* is the acquisition, evaluation, and appropriate application of relevant health information that allows consumers to communicate about health, make health decisions, and use health services [11,32].

Although this model of eHealth literacy is not inclusive of all factors that may influence the use of eHealth (e.g., knowledge of the social and cultural norms involved in participating in a support forum), it is our contention that these six literacy types constitute the set of core skills and knowledge domains.

We selected a second model that explains variation in task performance along an increasing continuum of cognitive demands. Bloom’s taxonomy is a well-known taxonomy developed in 1956 and was revised and updated in 2002 [33]. The taxonomy classifies levels of intellectual behavior in learning and has been applied to develop educational objectives and curriculum, assess learning, and create test items [33]. The cumulative hierarchy structure requires achievement of a prior skill before acquiring the next dimension of complexity, but the boundaries between these levels are not rigid. These six cognitive process dimensions are defined [34] as follows:

- *Remembering* is retrieving, recognizing, and recalling relevant knowledge from long-term memory.
- *Understanding* includes constructing meaning from oral, written, and graphic messages through interpreting, classifying, summarizing, inferring, comparing, and explaining.
- *Applying* involves using knowledge to execute a procedure.
- *Analyzing* comprises breaking material into constituent parts, and determining how the parts relate to one another and to an overall structure or purpose.
- *Evaluating* involves making judgments based on criteria and standards.
- *Creating* consists of putting elements together to form a coherent or functional whole in a new pattern or structure.

An overlay of Bloom’s taxonomy across the six eHealth literacies provides a framework to characterize and describe the different levels of cognitive demands within each of the six facets of eHealth literacy. It provides a structure to the analysis of human performance on eHealth tasks, allowing a differentiation of cognitive processes as well as of level of knowledge and skill.

The aim of this study was to characterize the constituent elements of eHealth literacy in performing tasks. The hypothesis was that this method can be used to elucidate the barriers to effective task performance.

## Methods

### Overview of the Framework and Method

**Figure 1:**
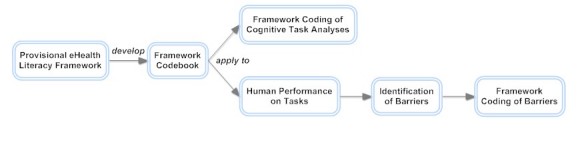
Process of employing a framework to characterize eHealth demands and barriers.

The framework can be expressed as a matrix with the six facets of eHealth literacy along one axis and the six levels of complexity along the other axis, resulting in 36 combined categories. In our framework, we further separated the category of traditional literacy and numeracy into reading, writing, and numeracy and analyzed each separately, as shown in Table 2, such that there are a total of eight different literacy types. In our preliminary application of the framework, it was evident that this revision was necessary to achieve sufficient level of detail for analysis. We defined the criteria for each of the cells through an iterative process of review and adaptation, drawing on evidence from peer-reviewed articles discussing eHealth and each type of literacy. This matrix of eHealth literacy and complexity definitions constituted the framework and codebook, providing the foundation for analysis. The framework coding can be used in two complementary ways. As Figure 1 shows, we employed a CTA and used it to characterize the demands of eHealth tasks with reference to specific tools. We also used the same categorical scheme to describe human performance on these tasks. The basis of the methodological framework involved coordinating task analysis and analysis of human performance.

**Table 2:** Framework shown as a matrix of literacy types and cognitive complexity levels

Literacy type	Increasing levels of cognitive complexity (Bloom’s taxonomy)					
	Remembering	Understanding	Applying	Analyzing	Evaluating	Creating
Computer						
Information						
Media		*The contents of this table are intentionally left blank. This table illustrates the structure of the framework coding tool, which can be used by researchers to map skill demands to the corresponding framework code in each cell of the table.*			
Reading					
Writing					
Numeracy					
Science					
Health									

### Application 1: Cognitive Task Analysis

To characterize eHealth literacy demands, we employed CTA, a cognitive engineering method that decomposes a task to uncover knowledge, goal structures, thought processes, and strategies underlying task completion [35,36]. Expert analysts carried out CTA by performing the task themselves, eliciting both information-processing demands of a task and the kinds of domain-specific knowledge required [37]. In the study of health technologies, CTA is most commonly used to study system usability, devise training protocols, or analyze technology-mediated work [38]. We applied CTA in a novel application, to characterize the actions, either behavioral or cognitive, and the knowledge needed to execute an eHealth task. For each task, CTA was used to enumerate the action and knowledge steps used to complete the specified task and to identify the constituent skills required to complete each step. Next, the codebook was used to select the types of literacy that describe the knowledge used in each step. We then determined the kinds of cognitive operations involved in the task that would provide us with a complexity level. For example, a step may require reading a text passage in order to follow the directions in the passage. To apply the framework code, we first identified that this step requires *reading literacy*, and then determined that reading is required at the *a* *pplying* level of complexity to use the information in the passage appropriately. The step also required *information literacy* at the *u* *nderstanding* level of complexity to be able to meet the appropriate information need while reading the passage. Most steps require more than one type of literacy.

In prior work, we illustrated the application of the framework analysis with CTA of three information-seeking tasks [22]. When applied to eHealth tasks, the framework provided illuminating representations of a task, displaying the configurations of eHealth literacy and cognitive demands for each task. The preliminary findings suggested that the approach enabled deeper exploration of the complex relationships and interactions of the different types of literacy. Our current research explored further applicability of the framework by applying the approach to a new task category, decision making, and to a wider range of health domains. We also applied the method to analysis of human performance and explored how the framework elucidates and diagnoses barriers encountered.

### Application 2: Analysis of Human Performance in Task Completion

In the second step of our method, we recruited 20 users to perform the same tasks and observed their performance. These individuals were active computer users but had no previous experience using the website employed in the study, the Consumer Reports Health website (http://www.consumerreports.org/health), a resource that helps consumers make evidence-based decisions related to health issues.

Participants were asked to verbalize their thoughts (a think-aloud protocol) while completing the task and to explain their answers. The think-aloud protocol can reveal any hesitation, confusion, or misunderstanding while completing the task [39]. It can also reveal insights into reasoning and decision-making processes [40]. While the participants were completing the task, guidance was provided when necessary to help them complete a task or to reroute them from a potentially fruitless path. Each session was audio recorded, and we used Morae 3.1 video-analytic software (TechSmith Corporation, Okemos, MI, USA) to capture all actions on the computer screen.

A step-by-step analysis of the participants’ performance was done based on the audio recording, video capture, and notes taken during observation of the session. The measures employed were (1) accuracy of response to each question in the task, and (2) any barriers encountered at each step toward completing the task. Task responses were scored according to a scoring scheme comprising specific criteria defining scores of 0 (incorrect), 1 (partially correct), or 2 (correct). In our analysis, barriers were defined as events where participants struggled and may have been unable to make progress in the task or may have required some problem-solving steps before moving forward in the task. Barriers may be indicated when participants required prompts, asked questions, or made errors. A prompt was noted if the researcher provided some verbal assistance to participants, such as directing them to appropriate information or reminding them about the next step of the task. Questions occurred when participants asked a question, expressed confusion, or requested guidance from the researcher. An error was documented if there was a misstep or misinterpretation of information or system response, such as misunderstanding search results. For each barrier event, the framework coding could be applied to categorize the nature of the participant’s problem in terms of a type of literacy. For example, difficulty with scrolling would be categorized as difficulty with a computer literacy skill, whereas struggling with text passages would be categorized as difficulty with reading skills. We also matched each event with the corresponding step in the task completion process in which it occurred.

### Example of Applying the Framework and Method

We applied these methods to the analysis of a particular task that required a series of information-seeking and decision-making steps. We selected the Consumer Reports Health website because, in our judgment, it is a high-quality and well-designed site that reflects a genuine understanding of consumers’ needs. The task question (see Textbox 1) asked users to consider criteria comparing three different hospitals, demonstrate understanding of the information, and interpret the evidence presented.

**Task Question Requiring a Series of Information-Seeking and Decision-Making Steps**

In the Doctors & Hospitals page, read the article “How-to guide to choosing a hospital” which can be found at the bottom of the page.

Look up the hospital ratings for **all** hospitals in the New York, NY region.

Next, on the ratings page, *use the Compare feature* to compare New York Presbyterian Hospital, Lenox Hill Hospital, and Bellevue Hospital Center.

- Identify the hospital that is least aggressive on the “Aggressive or Conservative” scale.
  What do these ratings of “Aggressive or Conservative” tell you about the hospital?
- Identify the hospital with the highest “Average Cost to Patient”.
- Of these 3 hospitals, select the hospital that you would want to go to for a surgical procedure, and discuss what criteria are most important in your decision.

**Figure 2:**
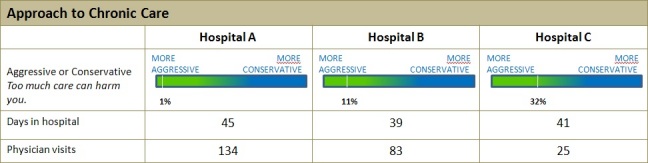
Representation of the aggressive/conservative scale. The rows are as they appear in the actual table on the webpage except for the top row, which is included for clarity.

Figure 2 shows a representation of the aggressive/conservative scale needed to interpret the “aggressive or conservative” continuum. These rows were extracted from a table on the pertinent Consumer Reports Health webpage. The aggressive-to-conservative continuum is one way in which the Consumers Union rates its hospitals. Hospitals that keep people with chronic diseases hospitalized for more days during the last 2 years of their lives are rated as aggressive. Hospitals that provide the least amount of doctor’s visits and shorted hospitalizations in those final years of life are considered conservative. We used example tasks to explore the reliability of the framework coding scheme. Two different raters used the codebook to classify task demands on two different tasks: an information-seeking and a decision-making task. The raters later used the codebook to also classify the barriers encountered by a subset of three different participants. For each type of coding, interrater reliability was assessed on two different dimensions of the coding: (1) type of literacy, using Cohen's kappa, and (2) level of cognitive complexity, using Spearman correlation coefficient. The assignment of a cognitive complexity code cannot be coded independent of literacy type. Therefore, cognitive complexity was calculated on the subset of codes in which both raters reached agreement on the literacy code.

## Results

To illustrate the application of the framework and method, we present the results from the analysis of the task question asking about hospital ratings.

### Application 1: Cognitive Task Analysis

Interrater reliability was calculated for coding of the CTA. Cohen's kappa for literacy was .91 and Spearman correlation coefficient for cognitive complexity was .92, suggesting high levels of agreement for both dimensions. Table 3 shows the CTA of an excerpt of this task (steps 10–16 of the entire task), from the steps for selecting the three specific hospitals for comparison to interpreting the aggressive or conservative scale.

**Table 3:** Application of the framework coding to steps 10–16 of the task

Step	Skills and knowledge required to complete step	Framework code from CTA^a^
10	Recognize the results page as a table of hospitals and their ratings. Scroll to see whole table.	Computer 3, information 4, numeracy 4, reading 1
11	Recognize the “compare” feature, and that checkboxes for the desired hospitals are required to use this feature. Select the appropriate checkboxes for the three hospitals.	Computer 3, information 3, reading 2
12	Recognize results as a table of the three selected hospitals with their detailed ratings. Scroll to see whole table.	Computer 3, information 4, numeracy 4, reading 2
13	Scroll to locate the “aggressive or conservative” row in the table. Interpret and understand the labels for the aggressive/conservative scale.	Computer 3, information 4, numeracy 4, reading 2
14	Identify the least aggressive rating and answer the information need.	Information 5, numeracy 4, reading 2, writing 2
15	Click on the “learn more” link. Find the newly opened window. Scroll down to find the text about aggressive/conservative hospitals. Read and understand text.	Computer 3, information 4, health 4, reading 3
16	Articulate understanding of what aggressive/conservative means.	Health 4, writing 3

^a^ Cognitive task analysis, by increasing complexity: 1 = remembering, 2 = understanding, 3 = applying, 4 = analyzing, 5 = evaluating, 6 = creating.

Completing these series of steps required the participant to navigate to the table, locate the relevant information, and interpret the data in the table. The aggressive/conservative scale (see Figure 2) corresponds to step 13 in Table 3. Each step was coded with the corresponding framework codes that describe the eHealth literacy and complexity level used to complete that step. For example, step 10 required a combination of four types of eHealth literacy: (1) information literacy at the analyzing level of complexity (information 4) was required to interpret and evaluate the results page, (2) numeracy at the analyzing level of complexity (numeracy 4) was required to interpret the results table, (3) computer literacy at the applying level of complexity (computer 3) was required to navigate and interact with the table, and (4) reading was required at the remembering level (reading 1) to make sense of the information in the table. The steps required different combinations of literacy types, ranging from a combination of two to four types of literacy. The highest complexity level of any eHealth literacy required was level 5, evaluating. Reading and information literacy were required for most of the steps in this excerpt and appeared more frequently than the other literacies.

Table 4 summarizes the results of coding the CTA for the entire task. For the whole task, reading was used most often, in 18 of the 20 steps (90%). Information literacy (17 of 20 steps) and computer literacy (15 of 20 steps) were also used often. The frequent use of these skills suggests that they are essential to completing the task and are useful skills to promote among health care consumers. Media literacy was not required for any steps in this task given that the website was already selected as part of the task. Information literacy was required at level 5 (evaluating) for two of three questions, suggesting that high levels of information literacy are necessary to be able to carry out all components of this task. As this task was primarily an information-seeking task, it is not surprising that information literacy was required frequently and at high levels of cognitive demand. Numeracy was required at level 4 (analyzing) for all three questions, to understand the information in different representational formats and to interpret the data in the table of hospital ratings. Question C was the only question to require science literacy, which was used to weigh evidence in making a decision about selecting a hospital based on the criteria presented. Question C also required the most skills at level 5 (evaluating), for two different types of literacy, suggesting that it had the highest complexity demands across the whole task. Question B was the only question to require any skills at level 2 (understanding); Question B had the lowest complexity demands relative to the other questions. The highest complexity level required across the whole task was level 5 (evaluating).

**Table 4:** Summary of task demands from cognitive task analysis

Literacy type	Question A	Question B	Question C	Whole task
Media	0%^a^	0%	0%	0%
	No complexity	No complexity	No complexity	No complexity
Computer	50%	50%	50%	75%
	Applying (3)	Applying (3)	Applying (3)	Applying (3)
Health	50%	0%	100%	35%
	Analyzing (4)	No complexity	Analyzing (4)	Analyzing (4)
Information	75%	100%	50%	85%
	Analyzing (4)	Evaluating (5)	Evaluating (5)	Evaluating (5)
Reading	75%	100%	50%	90%
	Applying (3)	Understanding (2)	Applying (3)	Applying (3)
Writing	50%	50%	50%	20%
	Analyzing (4)	Understanding (2)	Evaluating (5)	Evaluating (5)
Numeracy	50%	100%	50%	30%
	Analyzing (4)	Analyzing (4)	Analyzing (4)	Analyzing (4)
Science	0%	0%	100%	10%
	No complexity	No complexity	Applying (3)	Applying (3)
Total number of steps	4	2	2	20^b^

^a^ For the task, the following is displayed: the proportion (percentage) of steps that use that eHealth literacy and the highest level of cognitive complexity used in that literacy (number and complexity level).

^b^ Total number of steps for whole task includes a series of 12 navigational steps leading up to questions A, B, and C.

### Application 2: Analysis of Human Performance in Task Completion

The framework coding was then applied to the task performance. Interrater reliability was calculated for the coding of task performance. Spearman correlation coefficient for cognitive complexity was .88, suggesting high agreement. Cohen's kappa for literacy was .68, suggesting lower but sufficient agreement to meet the minimum standard. The results from a single user are displayed in Table 5.

**Table 5:** Mapping the framework coding for steps 10–16 to a participant’s performance on the task

Step	Skills and knowledge required to complete step	Framework code from CTA^a^	Events that indicate barriers	Framework code for barrier
10	Recognize the results page as a table of hospitals and their ratings. Scroll to see whole table.	Computer 3, information 4, numeracy 4, reading 1	Participant asks: “Aggressive or conservative scale—where’s that?” Participant is not on the correct page yet, needs to navigate to the next page first.	Computer 2, information 2
11	Recognize the “compare” feature, and that checkboxes for the desired hospitals are required to use this feature. Select the appropriate checkboxes for the three hospitals.	Computer 3, information 3, reading 2	Researcher prompts: “Use the ‘compare’ feature.”	Computer 3, information 1
			Error: participant clicks on “compare” without having selected the hospitals to compare.	Computer 3
			Researcher prompts: “In order to compare the three, you want to select all three together.”	Computer 3, information 2
12	Recognize results as a table of the three selected hospitals with their detailed ratings. Scroll to see whole table.	Computer 3, information 4, numeracy 4, reading 2	No barrier encountered during this step.	None
13	Scroll to locate the “aggressive or conservative” row in the table. Interpret and understand the labels for the aggressive/conservative scale.	Computer 3, information 4, numeracy 4, reading 2	Participant confused by the multiple parts of the task question. Researcher prompts: “Look at this part of the question first.”	Information 1
			Participant scrolls up and down, and finds the aggressive/conservative scale. Starts to read ahead to the next question. Researcher prompts again: “Try this question first—the hospital that is least aggressive.”	Information 1
			Participant asks: “Where does it tell you which is least or most aggressive/conservative? In this area here?” (pointing to the scale).	Information 2, numeracy 4
14	Identify the least aggressive rating and answer the information need.	Information 5, numeracy 4, reading 2, writing 2	Participant stares at scale, confused. Researcher prompts: “What do you think the scale is telling you; how are you reading the scale?”	Numeracy 4
			Participant is very confused by the scale, and answers: “The one that is more conservative is 32%, Bellevue. Least aggressive, Lenox Hill? I’m trying to understand this.” (incorrect)	Numeracy 4
15	Click on the “learn more” link. Find the newly opened window. Scroll down to find the text about aggressive/conservative hospitals. Read and understand text.	Computer 3, information 4, health 4, reading 3	Participant is unsure how to approach the next question. Researcher rewords the question and explains what the question is asking.	Information 1, reading 2, information 2, reading 2
			Participant clicks on the “learn more” link and scrolls down the page, but cannot find the relevant text. Participant scrolls past the relevant passage. Researcher prompts: “You just missed the description on the page.”	
16	Articulate understanding of what aggressive/conservative means.	Health 4, writing 3	Participant reads the text passage, then answers: “More doctors visit overall for aggressive/conservative care...fewer days in the hospital.” (incorrect)	Health 3

^a^ Cognitive task analysis, by increasing complexity: 1 = remembering, 2 = understanding, 3 = applying, 4 = analyzing, 5 = evaluating, 6 = creating.

This participant scored low on this task, earning 2 out of a total of 6 possible points. The participant encountered 18 barriers while completing this task. In step 10, the participant was looking for a specific piece of information but was on the wrong page; this barrier can be attributed to problems or deficiencies associated with information and computer literacies. In step 14, the participant was confused by the scale and provided an incorrect answer due to misinterpretation of the information presented in the aggressive/conservative scale. This barrier reflects a struggle with numeracy because the participant demonstrated an understanding of numbers as evidenced by the ability to draw inferences about the scale, but was unable to *apply* the knowledge and *analyze* it in different representational formats. Then in step 16, the participant provided an incorrect answer. The participant was unable to read, interpret, and analyze the health text to extract an accurate description of the terms aggressive and conservative as used in this context; this barrier reflects a struggle with health literacy. The participant required several reminders or explanations of task questions, in steps 11, 13, and 15. These reminders and explanations indicated information literacy barriers, reflecting a lack of recognition and understanding of the nature of the information need.

### Summary Results From 20 Participants

A summary of 20 users’ task performance results are presented to illustrate the aggregate measures obtained and potential analyses that can be performed using our approach. The users were recruited from the Union Settlement Association and the Columbia Community Partnership for Health Center in New York, NY.

Participants recruited were adults between 18 and 65 years of age; all had basic proficiency with computers and the Internet. A total 14 of 20 (70%) of participants were female, most reported annual incomes below US $30,000, and a majority of participants reported their race as African American or Hispanic. Participants had a range of education backgrounds, with 7 participants reporting high school education, 7 having a college degree, and 6 with a graduate degree.

**Figure 3:**
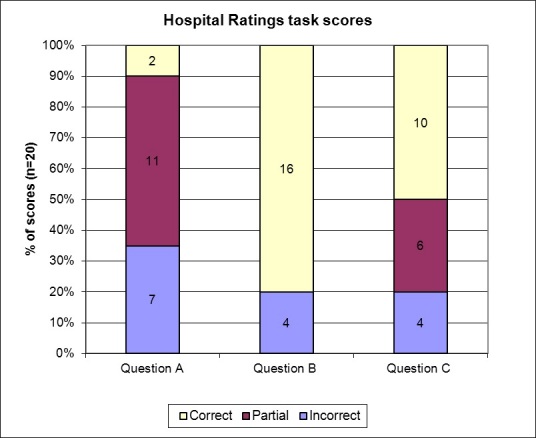
Hospital ratings task: distribution of participants’ scores on questions A, B, and C, and average (Avg) scores for each question.

As Table 4 describes, Question B had the lowest literacy and complexity demands relative to the other questions. Figure 3 shows that participants scored highest on this question, with 16 out of 20 correct answers. Participants struggled most with question A, with only 2 correct answers, and 11 partial answers. Although question C had the highest complexity levels of cognitive demands, 10 out of 20 participants (50%) answered this question correctly. Each question varied in terms of domain knowledge, complexity, and types of demands. Scores merely provide a snapshot of user task performance. Although we can use the scores to compare and contrast task performance across the different task questions, analysis of the barriers impeding task performance can yield additional insight into the resulting participant scores.

**Figure 4:**
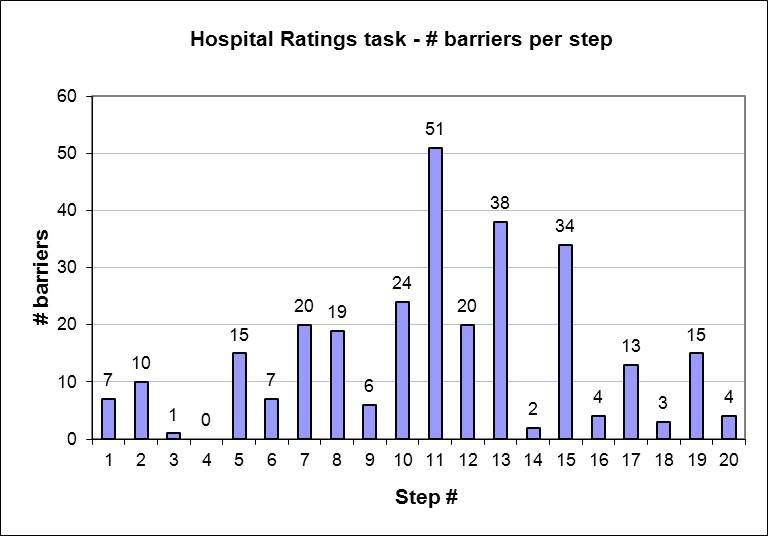
Number of barriers encountered by participants in each step, with labels for the steps that constitute questions A, B, and C.

Figure 4 shows the number of barriers encountered by all participants for each step. The most barriers were encountered in step 11, with a total of 51 barriers encountered. This step required users to make the appropriate selections in order to compare the different hospitals selected. Most of the barriers on this step stemmed from unfamiliarity with making the appropriate selections using checkboxes, reflecting inadequate computer literacy. Users encountered a high number of barriers at steps 13 and 15 as well. These steps are both constituents of question A, on which participants scored the lowest of the three questions. This aggregate analysis revealed the steps in which users experienced the most difficulty and exemplifies the patterns of barriers encountered in carrying out those problem steps.

**Figure 5:**
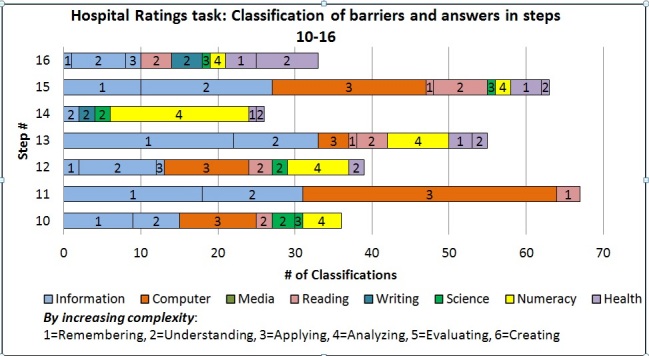
Barriers encountered by participants (n = 20) in steps 10–16, categorized by literacy (color in legend) and complexity level (number in the graph).

We aggregated the types of barriers encountered by users in a manner similar to the analysis in Clark et al [41], which provided cumulative descriptors of the component barriers encountered across a set of steps and tasks. Figure 5 presents the classifications of literacy type and cognitive demands of barriers encountered in task performance. The same excerpt of steps (steps 10–16) was depicted as in Table 3. Most of the barrier classifications in these steps are due to barriers with information and computer literacy. Step 13, which required understanding question A, caused many barriers at levels 1 (remembering) and 2 (understanding) within information literacy. These barriers primarily involved struggling to identify and interpret the information need. Step 14, which required locating and interpreting the aggressive/conservative scale, led to many numeracy level 4 barriers (analyzing based on representation). Step 15 asked users to describe the meaning of aggressive/conservative in the context of hospitals and health care, and users struggled with finding resources to meet this information need. These barriers are reflected by the majority of barriers being information literacy and computer literacy barriers. Step 16 reflected many health literacy as well as some writing barriers; users struggled with understanding, interpreting, and articulating aggressive/conservative in their own words. The majority of barriers fell in the lower ranges of cognitive demands (levels 1–4). The task demands also required mainly literacies at these lower levels. The patterns of barrier types as revealed by the coding reflected the nature of the task demands and provided insight into the types of barriers that participants encountered.

Overall, within the hospital ratings task, users scored highest on question B and encountered the most barriers in question A. The barriers identified reflected that users struggled primarily with information literacy, computer literacy, and numeracy skills in answering the question and completing the tasks.

## Discussion

In this research, we adapted and integrated two existing theoretical models relevant to the analysis of eHealth literacy into a single framework to systematically categorize and describe task demands and user performance on tasks needed by health care consumers in the information age. The method derived from the framework is applied to (1) code task demands using a CTA, and (2) code user performance on tasks. The analysis shows that the framework can be used to classify task demands as well as the barriers encountered in user performance of the tasks. Our approach can be used to (1) characterize the challenges confronted by participants in performing the tasks, (2) determine the extent to which application of the framework to the CTA can predict and explain the problems encountered by participants, and (3) inform revisions to the framework to increase accuracy of predictions. In this study, we used the methods to document a range of literacy-related barriers that affected performance on eHealth tasks.

The study found that 20 participants experienced some difficulty completing most tasks on a website designed for consumers without some assistance. The most frequent barriers encountered by our sample were challenges with information literacy and computer literacy skills. Specific examples of frequent barriers encountered are struggling with the ability to understand and successfully act on information needs, to interpret a graphical representation of a severity scale, and to effectively use checkboxes to make selections. Conversely, some activities in which we had predicted barriers were discovered to be easier than anticipated. Evaluating health information to inform decisions can be complex and challenging, but users scored well on the question with a decision point.

There is little existing research that systematically analyzes the combined set of eHealth skills needed to attain proficient performance. Other investigators have expanded the scope of health literacy to describe the combinations of skills needed to interact effectively with health information [42] but did not consider technology-related skills, such as computer literacy, that are a core part of eHealth literacy. Our results largely echo findings in prior health numeracy research that users often struggled with interpreting graphical representations of numerical information, which may constitute significant consumer barriers [29]. Our findings also support recommendations to develop tools that aid health care consumers in understanding complex health concepts and to use the information to inform a decision [43]. Usability studies take a similar approach in breaking down task demands to analyze user task performance. Our method is consistent with usability findings that a granular approach to task analysis is essential to reveal potential barriers and inform design improvements, particularly for novice users [44].

### Limitations

We view the framework as provisional and subject to more comprehensive validation and elaboration. This will necessitate a larger-scale study with a greater sample size, a more diverse population, and a wider range of tasks. In addition, the participants in the study were not familiar with the Consumer Reports Health website and this may have influenced our findings. Familiarity with content, style, and affordances common to this site would have likely reduced some of the barriers that participants experienced. Further studies should include participants with varying degrees of experience with a particular website or technology.

The analyses in this paper focused on user competencies and did not take into consideration a range of issues, such as usability, or affordances and resources available within specific technology tools. In addition, the methods employed did not take into account individual motivation or attitudes toward technology. Similarly, this cognitive rational framework does not capture emotional and social factors that also play a significant role in decision making. It is well known that health literacy is a major public health issue in the United States affecting a substantial segment of the population [11]. In general, a multitude of environmental and societal factors, such as differential access to the eHealth tools, influence the productive use of technology in health-related contexts. Although these individual and social factors significantly influence task performance, our leading-edge hypothesis is that eHealth literacy is a distinct construct and an important one in consumer health informatics.

As previously described in Table 1, there are many different types of eHealth tools and eHealth tasks. The framework was illustrated using an example task on the Consumer Reports Health website. This website aims to present information simply and comparatively. Consumer Reports has been presenting unbiased and evidence-based comparisons in print form for many years. However, evidence in health is often complex and there may be alternative ways for rendering such information as comprehensible to individuals lower in eHealth literacy. The effective presentation of health evidence is a challenge that continues to plague most health communication and decision aid materials [45]. The website selection was sufficient for the purpose of illustrating the framework. It should be noted that the aggressive/conservative continuum scale is no longer used on the Consumer Union’s health site. Further exploration will apply the framework to a wider array of tasks, tools, and health domains.

### Further Development of the Framework and Analytic Method

Further studies are needed to determine whether the types of literacy described in this paper sufficiently cover the range of knowledge types that characterize eHealth competency. In addition, although Bloom’s taxonomy has an established history of characterizing cognitive dimensions of tasks in educational contexts, we cannot presuppose that the gradations of complexity will seamlessly transfer to eHealth. The results of this analysis suggest that it can be used meaningfully to differentiate and categorize cognitive demands for different literacy skills and can be used to approximate complexity in a range of eHealth tasks.

As discussed, the tasks used in the study did not delve deeply into media, science, and to some extent health literacy. As health consumers choose what resources to use, media literacy will loom large. We anticipate that our methods will be adequate to model the skills and knowledge needed to demonstrate media literacy competency. The problems associated with low health literacy are well documented [11]. Science literacy is a multifaceted construct, and there is ample evidence to suggest that problems associated with science literacy are equally profound. The general public in the United States and other countries have an impoverished understanding of science [46]. Norman and Skinner [23] situate scientific literacy in a broader context, defining it as “understanding of the nature, aims, methods, application, limitations, and politics of creating knowledge in a systematic manner.” The framework employs a CTA approach that places a strong emphasis on skills and action. This may not capture other dimensions of science literacy such as understanding biological mechanisms of disease and critical appraisal of the scientific process. These aspects come into play in situations such as when an individual must understand the consequences of a therapeutic regimen or decide whether to enroll in a randomized controlled clinical trial. Clearly, we would need a broader array of concepts and a richer set of representations than those offered by the CTA stepwise analytic method to model such knowledge and causal inferences associated with its application in the context of health.

The proposed framework provides a basis for the development of an eHealth competence model. Such a model would yield insight into the specific skills and knowledge needed to perform at a proficient or higher level on system-specific instances of eHealth tasks, such as seeking information about hypertensive therapies on the WebMD site. The current set of framework-based methodological tools lends greater utility to the consumer health research community than to communities of practitioners and designers. Applying this method is time intensive and requires moderate expertise in the areas of cognition and human–computer interaction. We anticipate that the framework would give rise to simpler, more specific instruments (for example, in the form of a set of questions or heuristics) that could measure eHealth demands for a particular task and population as realized in a particular system or device. An analogy would be Nielsen’s heuristic evaluation method [47], which has made it possible for teams of developers to conduct basic usability evaluations without extensive training or prohibitive time commitments.

With further investigation, we envision that the framework and analytic approach can be a potentially powerful generative research tool for development of design guidelines of computer-based tools, evaluation heuristics, task-based eHealth literacy assessment, and educational objectives to increase consumer eHealth skills. For example, the framework could form the basis for development of a matching algorithm to identify appropriate tools for users with different skill sets. In particular, this framework and analysis method can be used with health care consumers with low eHealth skills to better understand barriers and to develop educational media or other mediating tools to facilitate engagement with and benefit from eHealth. Barriers fall on a continuum ranging from routine abilities (recognizing how to use widgets) to complex conceptual challenges (deriving inferences from health text). The proposed framework systematically characterizes eHealth barriers, which in turn enables more precise definition within the solution space of methods to overcome those barriers.

### Conclusions

In our view, this framework provides a systematic and potentially rigorous approach for analyzing eHealth competencies, which is a challenge of considerable complexity and great significance. Advances in technologies, such as Web 2.0 and social networking functionalities, offer new and ever-changing modes for consumers to interact with and manage health information. In the current environment where eHealth interventions are being developed without a thorough understanding of the consumers, efforts, and resources can be better focused to improve adoption and use rates as well as benefit from use. Unfortunately, these barriers disproportionately affect those who are most vulnerable and may actually serve to exacerbate disparities rather than bridge them. There is no doubt that consumers will be expected to assume a greater role in their health management in coming years, and low eHealth literacy will continue to be a barrier to productive participation. Progress in eHealth research will be integral to the success of consumer health applications and for reducing barriers to the use of those applications.
